# Comprehensive proteomic profiling of plasma-derived Extracellular Vesicles from dementia with Lewy Bodies patients

**DOI:** 10.1038/s41598-019-49668-y

**Published:** 2019-09-16

**Authors:** Ana Gámez-Valero, Jaume Campdelacreu, Ramón Reñé, Katrin Beyer, Francesc E. Borràs

**Affiliations:** 1 Department of Pathology, Hospital Universitari and Health Sciences Research Institute Germans Trias i Pujol, Universitat Autònoma de Barcelona, Badalona, Barcelona Spain; 2 REMAR-IVECAT group, Health Sciences Research Institute Germans Trias i Pujol, Badalona, Barcelona Spain; 3 Nephrology Service, Hospital Universitari Germans Trias i Pujol, Badalona, Barcelona Spain; 4 Department of Cell Biology, Physiology and Immunology, Universitat Autònoma de Barcelona (UAB), Barcelona, Spain; 5 Department of Neurology, Hospital Universitari de Bellvitge, L’Hospitalet de Llobregat, Barcelona Spain

**Keywords:** Mass spectrometry, Proteomics, Cellular neuroscience, Diagnostic markers

## Abstract

Proteins and nucleic acids contained in extracellular vesicles (EVs) are considered a feasible source of putative biomarkers for physiological and pathological conditions. Within the nervous system, not only neurons but also other brain cells are able to produce EVs, which have been involved in their physiological processes and also in the development and course of several neurodegenerative diseases. Among these, dementia with Lewy bodies (DLB) is the second cause of dementia worldwide, though most cases are missed or misdiagnosed as Alzheimer’s disease (AD) due to the important clinical and pathological overlap between both diseases. In an attempt to find reliable biomarkers for DLB diagnosis, our group characterized the proteome of plasma-derived EVs from DLB patients compared to aged-matched healthy controls (HCs) using two different proteomic LC-MS/MS approaches. Remarkably, we found that gelsolin and butyrylcholinesterase were differentially identified between DLB and HCs. Further validation of these results using conventional ELISA techniques, and including an additional group of AD patients, pointed to decreased levels of gelsolin in plasma-EVs from DLB compared to HCs and to AD samples. Thus, gelsolin may be considered a possible biomarker for the differentiation between DLB and AD.

## Introduction

Extracellular vesicles (EVs) are nanovesicles of multiple sizes surrounded by a lipidic bilayer^1,2^ and released by almost all cell types - including cells from the central nervous system-, that play important roles in intercellular communication, immunomodulation or inflammation^2–4^. The cargo contained in EVs from different sources has been studied intensively during the last years as possible source of biomarkers as it is considered to reflect the state of the producing cells^5–7^. In neurodegenerative diseases, EVs have been described as one of the main effectors in the pathogenesis of several disorders, including proteinopathies like Alzheimer’s disease (AD), Parkinson’s disease (PD) or Multiple System Atrophy, all of them characterized by the deposition of misfolded proteins in defined brain areas^3^. Therein, their role in the spread of α-synuclein during PD progression has already been assessed^8,9^; also, EVs are implicated in the activation of glial cells and they act as neuron-glia communication mediators^10^. In addition, some studies have reported the presence of tau in EVs as aggregation effectors in AD^11,12^; and their implication in the lysosomal impairment in neurons or in the induction of neuronal apoptosis by astrocytes during AD course has also been described^10,13^.

Among neurodegenerative diseases, dementia with Lewy bodies (DLB) is the second cause of dementia worldwide, accounting for around the 25–30% of all dementia cases^14^. Together with PD, it is considered a synucleinopathy, and it is characterized by the deposition of α-synuclein in the cerebral cortex^15^. However, DLB can also present pathological hallmarks of AD such as tau deposits or β-amyloid plaques throughout the brain^16,17^. This pathological overlap between the three most common neurodegenerative disorders is also accompanied by a clinical overlap, thus hindering DLB diagnosis. Hence, many DLB patients are clinically missed or misdiagnosed as AD or PD and, consequently, lacking or even adverse response to treatment is common^18^. Attempting to solve this issue, during the last decade research has been focused on finding specific biomarkers that could better characterize these heterogeneous and complex diseases^19^. In this scenario, EVs have emerged as a perfect source of specific biomarkers as their protected content could reflect the physiological and pathological state of a specific tissue, organ or cell type^20^. Furthermore, as they can be isolated from several body fluids such as urine, saliva, blood or milk, their analyses only require a minimally invasive intervention^20–22^.

Until now, no study has examined the proteomic profile of plasma EVs from DLB patients. In the current study, we used two different proteomic approaches to characterize the proteomic profile of plasma-derived EVs from DLB patients and age-matched healthy controls in order to identify reliable biomarkers for this disease in a minimally invasive fashion. Despite the expected differences observed between the two processing/analysis approaches, gelsolin appeared as a promising biomarker from plasma EV in order to differentiate DLB from HCs. Additional validation using conventional ELISA techniques further confirmed this hypothesis and, interestingly, pointed to gelsolin as a putative marker to discriminate between DLB and AD patients.

## Materials and Methods

### Patients

A cohort of DLB patients (n = 19; age range 57–86 years; mean 71.8 years; male:female ratio 3:2), and age- and gender-matched healthy controls (HCs) (n = 20; age-range 61–78; mean 69.2 years; male:female 1:2), both from the Bellvitge University Hospital, (L’Hospitalet de Llobregat, Barcelona) were recruited. Diagnosis of DLB patients was established according to the 2005 DLB Consortium criteria^23^ defining age at onset as the age when memory loss or parkinsonism was first noticed by relatives. An additional group of AD patients (n = 10; age range 65–85; mean 73.9; male:female ratio 3:2; Global Deterioration Scale score 4.3 ± 1.2) was also enrolled for the validation phase of the results. AD diagnosis was assessed in the Neurology Department of the same hospital following the 2011-revised criteria from the National Institute on Aging and the Alzheimer’s Association^24^.

The applied protocol was approved by the “Germans Trias i Pujol” Clinical Research Ethics Committee and written informed consent was obtained from each subject according to the Declaration of Helsinki Principles^25^.

### Samples and blood collection

According to the International Society for Extracellular vesicles (ISEV) recommendations, peripheral blood was collected avoiding platelet contamination and activation^26–28^. In short, 2–3 mL of blood were discarded after vein puncture, and 15 mL of peripheral blood were collected per patient using a 21-gauge needle coupled to a butterfly device in sodium citrate pre-treated tubes (BD Vacutainer, New Jersey, USA) to avoid coagulation. After gently inverting the tubes 5–8 times, samples were processed within the first 2 hours following the collection. Serial centrifugations were applied in order to obtain platelet-free plasma. Briefly, blood was centrifuged at 500 × g for 10 min in order to remove most blood cells, and the supernatant was subjected to a second centrifugation at 2,500 × g for 15 min obtaining a platelet-enriched pellet and a platelet-free plasma supernatant. A final centrifugation step at 16,000 × g for 10 minutes in order to remove biggest particles was applied^29^. Samples were kept at −80 °C until EV purification.

### EV purification and characterization

Two millilitres of the purified centrifuged platelet-free plasma were used to isolate EVs by Size Exclusion Chromatography (SEC)^30–32^. Briefly, 20 mL of Sepharose-CL2B (Sigma-Aldrich, St. Louis, MO, USA) was stacked in a Puriflash column Dry Load Empty 12 g (20/pk) from Interchim (France)-Cromlab, S.L. (Barcelona, Spain) and after column preparation, 2 mL of plasma were loaded. Sample separation and elution by SEC was performed using filtered PBS as elution buffer. Thirty fractions of 0.5 mL were collected and analysed for the expression of tetraspanin specific EV-markers CD9, CD63, and CD81 by bead-based flow cytometry as previously described^22^.

Tetraspanin-positive fractions, as detected by mean fluorescence intensity (MFI) in the FACS analysis (Flow Jo software, Tree Star, Ashland, OR) were considered as EV-containing fractions. Cryo-electron microscopy was also applied to better characterize the isolated EVs. Protein concentration of each fraction was measured by absorbance at 280 nm in Thermo Scientific Nanodrop® ND-1000 (Thermo Fisher Scientific, Waltham, MA) and by bicinchoninic acid assay (BCA assay, 562 nm) (Thermofisher Scientific, Waltham, MA) before proteomic analysis.

### Mass spectrometry and protein identification

SEC-isolated EVs were analysed in two different sets. A first set included 6 DLB plasma-EVs and 6 HC plasma-EVs. A second set included 10 additional samples (5 DLB and 5 HCs). In both cases, individual samples were run separately. Two DLB and two HC samples were analysed in both sets, as internal controls.

For the first set, a volume of 500 µL of isolated-EVs was lyophilized and re-suspended in 6 M Urea 200 mM ammonium bicarbonate prior reduction, alkylation and digestion with LysC and Trypsin. Desalted samples were analysed by LC-MS/MS (LTQ-Orbitrap XL) with a C18 chromatography column (Nikkyo Technos NTCC-360/75-3-125) using gradients from 93% buffer A, 7% buffer B to 65% buffer A, 35% buffer B, in which buffer A was 0.1% formic acid in water and buffer B, 0.1% formic acid in acetonitrile. The instrument was operated in data dependent acquisition mode and full MS scans at resolution of 60,000 with detection in the Orbitrap. Following each survey scan, the top ten most intense ions were selected for fragmentation via collision-induced dissociation (CID) and acquired in the linear ion trap.

The second set of isolated vesicles was concentrated by ultrafiltration (instead of lyophilization) using 10 kDa cut-off Amicon Ultra devices (Merck Millipore, Darmstadt, Germany), and the PBS buffer was changed to 6 M Urea 50 mM ammonium bicarbonate. The samples were then run on a 10% SDS-PAGE gel that was stained with colloidal Coomassie blue. The acrylamide sections containing the protein mixtures were cut, washed, dehydrated and subjected to reduction and alkylation with 200 μl of 55 mM Iodoacetamide in 50 mM ammonium bicarbonate for 30 minutes, protected from light. They were digested with Trypsin. Obtained peptides were dried in a SpeedVac and stored at −20 °C until analysed by liquid chromatography-mass spectrometry in a linear ion trap Velos-Orbitrap mass spectrometer (Thermo Fisher Scientific, Bremen, Germany). Instrument control was performed using Xcalibur software package, version 2.2.0 (Thermo Fisher Scientific, Bremen, Germany). Digests were loaded onto a trapping guard column and eluted from the analytical column by using a mobile phase from 0.1% FA (Buffer A) and 100% acetonitrile with 0.1% FA (Buffer B) and applying a linear gradient from 5 to 35% of buffer B for 120 min at a flow rate of 300 nL/min. The LTQ Orbitrap Velos mass spectrometer was operated in data-dependent mode. The 20 most abundant ions were selected for CID fragmentation in the linear ion trap when their intensity exceeded a minimum threshold of 1000 counts, excluding singly charged ions.

**Figure 1:**
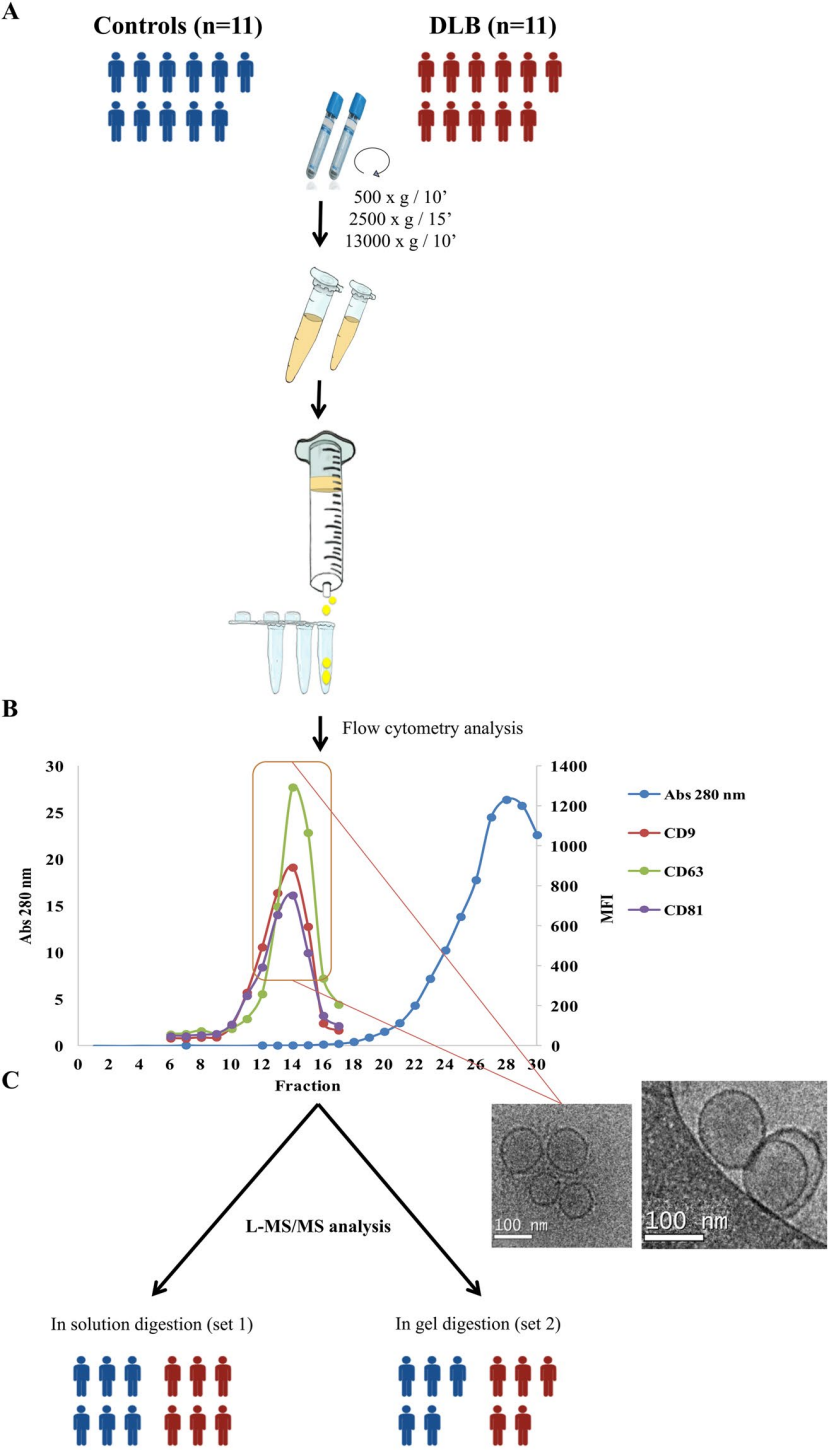
Workflow: Extracellular vesicles were isolated by SEC and submitted to two different shot gun proteomic approaches. (**A**) Two mL of platelet-free plasma obtained by differential centrifugation were loaded onto a sepharose column and vesicles were obtained by SEC. (**B**) Up to 30 fractions were collected and analysed for total protein content by Nanodrop and absorbance at 280 nm together with the presence of CD9, CD63 and CD81 by flow cytometry. **(C)** Highest MFI fractions for the three EV-markers were submitted to cryo-electron microscopy and to proteomic analysis. Blue group is identified as control cohort; red group identifies the DLB group. The figure was hand-drawn by collaborator Dr. Carolina Gálvez-Montón.

A schematic representation of the followed workflow is shown in Fig. 1.

### Data analysis

In both approaches, acquired data were analysed using Proteome Discoverer software (Thermo Fisher Scientific). For peptide identification, the data was searched against SwissProt Human database with the search engine Mascot (Matrix Science, London UK). The identified peptides were filtered using a mass tolerance of 10 ppmm, fragment tolerance of 0.05 Da, trypsin specificity (and LysC specificity, in the first set) with a maximum of 2 missed cleavages, cysteine carbamidomethylation set as fixed modification and methionine oxidation as variable modification. The significance threshold for the identifications was set to p < 0.05 and a minimum ion score of 20. The average of the area under the chromatographic peak for the three most intense peptides per protein was used as a measure of protein abundance.

An additional analysis of both sets of samples was performed using MaxQuant Software (version 1.6.0.1). Raw data from LC-MS/MS were analysed against the Uniprot human database (downloaded on 2^nd^ June 2017 from http://www.uniprot.org) as reference. Protein identification was performed taking into account a minimum peptide length of 7, FDR = 1%, minimum peptides per protein of 1 and minimum unique peptides per protein 0; minimum score for modified peptides of 40 and main search error of 4 ppm. Subsequent analyses, based on the logarithmized Intensity-based Absolute Quantification (iBAQ) values from MaxQuant analysis, were performed using Perseus software^33^ (version 1.6.0.2) and taking into account only proteins identified by at least 1 unique peptides, not identified only by reverse or only by site and removing potential contaminants, as described by our group^32^.

Venn diagrams were obtained using online tool http://www.interactivenn.net/ and FunRich analysis software and plasma-EVs related proteome was obtained from Vesiclepedia, Exocarta and EVpedia databases^5,6,34–36^. Using also FunRich and, corroborated with PANTHER Overrepresentation Test (release 20181106)^37^ using as reference list “Homo Sapiens (all genes in database)”, we performed Gene Ontology (GO) analysis for cellular component and molecular function.

### ELISA Assay

A preliminary validation strategy of the two proteins showing highest differences was performed by enzyme-linked immunosorbent assay (ELISA)^38^ in independent cohorts of DLB patient and controls together with an additional cohort of AD patients. A volume of 1.5 mL of isolated vesicles were ultrafiltrated using 100 KDa Amicon Ultra 2 mL (Merck) and the obtained volume was lysed with 100 mM Tris (ph 7.4) 2% NP40 detergent and submitted to a heat-thaw cycle. Pre-coated plates from ELISA kits for gelsolin (GSN; EKU04357) and for butyrylcholinesterase (BCHE; EKU02818) from Biomatik (Cambridge, Ontario, USA), were used following the manufacturer’s instructions.

### Statistical analysis

Data and results form ELISA assay  are presented as mean ± standard deviation (SD). Statistical analyses were performed using the Student’s t test. Differences were considered significant when p < 0.05.

## Results

### EV isolation and characterization

Plasma samples from two groups, DLB patients and age-matched HC individuals, were used for the isolation of EVs and submitted to proteomic analysis using two different approaches. All EV-isolations were performed by SEC (Fig. 1A), rendering a minimal-serum protein contamination in vesicle-enriched fractions as measured by Nanodrop (Fig. 1B). Identification of EV-presence was assessed by CD9, CD63 and CD81 staining by flow cytometry (Fig. 1B) and highest MFI fractions were considered as EV-enriched and pooled in 1.5 mL. Using cryo-electron microscopy, we checked for the presence and integrity of the characteristic bilayer round-sized structures in the pooled fractions as also shown in Fig. 1C.

### Proteomic analysis of vesicles-enriched fractions

**Figure 2:**
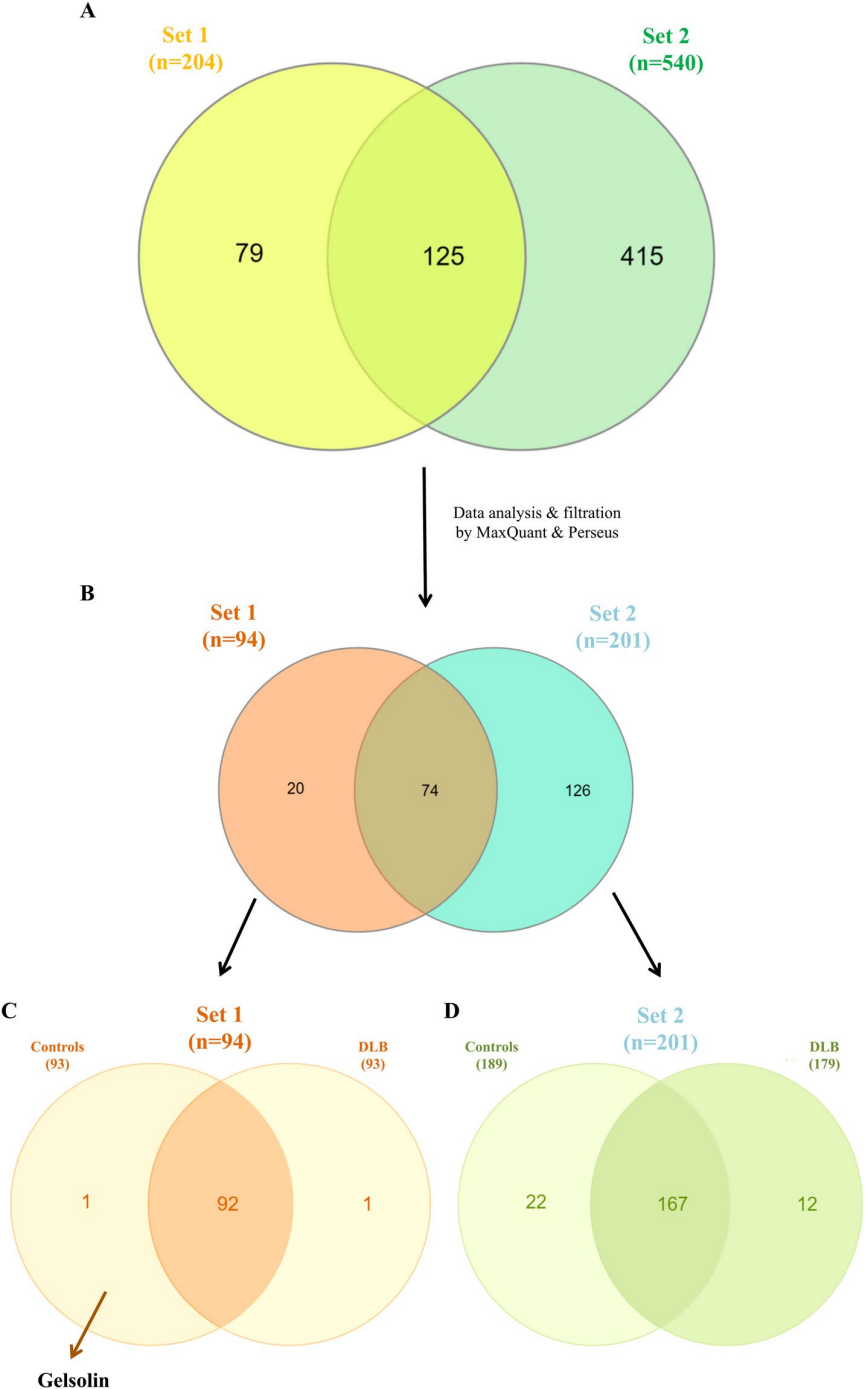
EV-proteomic qualitative characterization of samples from both sets. (**A**) Venn diagram showing the overlap of proteins detected among the two batches analysed after the shot gun approach and Mascot Engine identification. **(B)** Data were filtered by MaxQuant software and analysed by Perseus Software obtaining only the proteins identified with 1 or more unique peptides. **(C)** In set 1, 98% of the proteins overlapped between the two cohorts, controls and DLB. **(D)** In set 2, 167 proteins were common in control and DLB cohorts.

Isolated vesicles were subjected to the two different proteomic approaches. Lyophilized samples from the first set, composed of 6 DLB patients and 6 HCs, resulted in the identification of a total of 204 proteins with Mascot search engine (Fig. 2A). The analysis of the second set of 5 DLB and 5 HC samples by in gel digestion and using the same software resulted in a recovering of 540 proteins (Fig. 2A).

**Figure 3:**
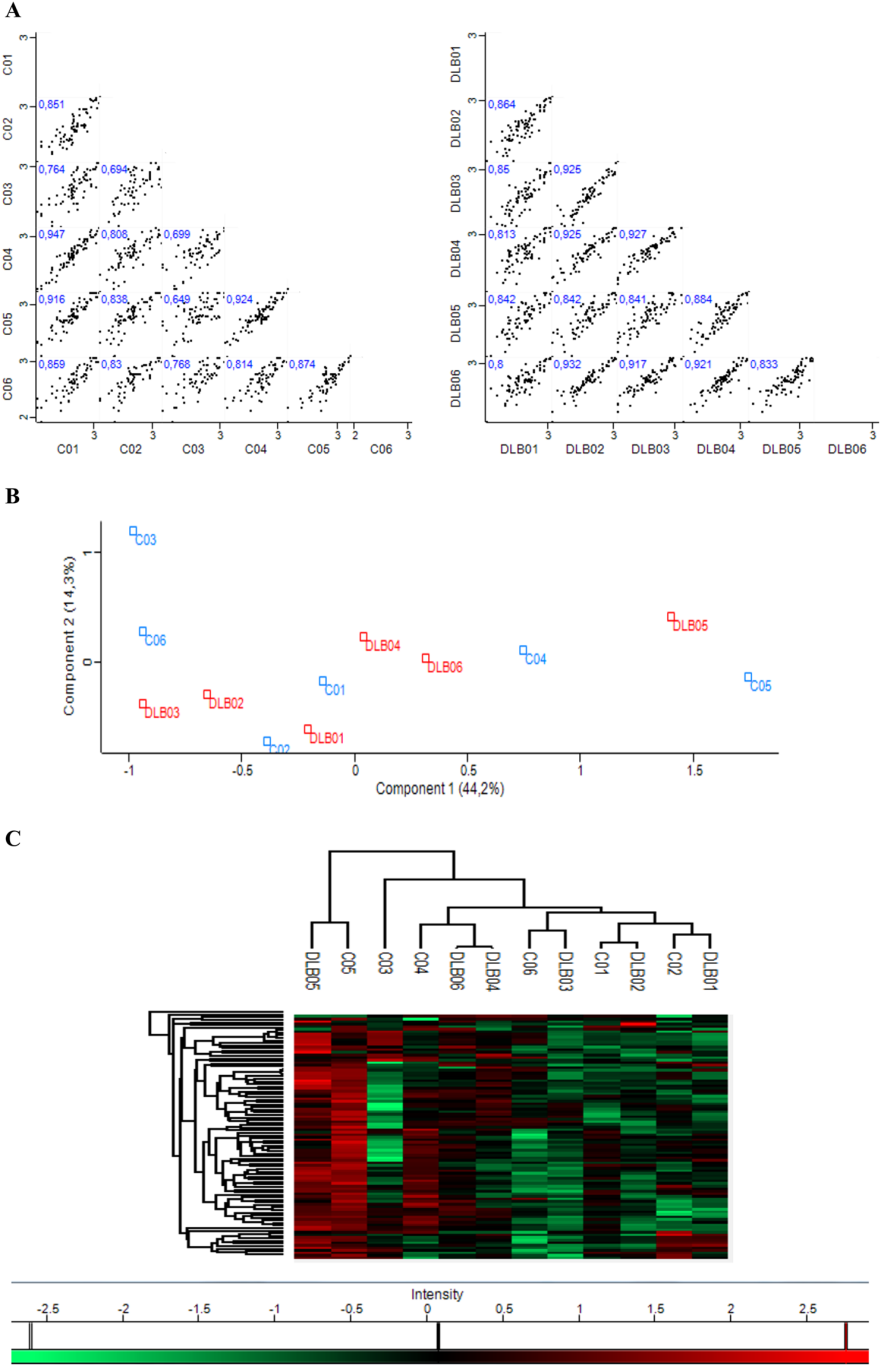
Similarities and differences between DLB and control cohorts from Set 1. (**A**) Multi-scatter plots were calculated to visualize intragroup similarities. Pearson correlation values are labelled on each plot. **(B)** Comparative protein content analysis of both cohorts by PCA showing components 1 and 2, which account for 44.2% and 14.3%, respectively; **(C)** Hierarchical clustering analysis with heat map of the 94 proteins (rows) and the samples (columns).

Focusing on the proteins covered in Set 1, after re-analysing the raw data by MaxQuant software and filtering them using Perseus software (removing possible contaminants and considering only proteins identified at least with 1 unique peptide), 94 proteins (46.1% of the initially covered) were considered for further analysis (Fig. 2B). Most of the proteins identified in this first set of analysis were present in both cohorts, DLB and HCs (Fig. 2C). However, two specific proteins were differentially identified in one cohort or the other. Gelsolin (GSN) was present in 5 of the 6 HC samples and in none of the DLB samples, and while in the DLB group, statherin (STATH) was present in one sample, it was not found among control samples (Fig. 2C). Perseus software identified an average of 79.2 ± 3.3 proteins per sample among the DLB-EVs, and 79.2 ± 6.7 proteins in HC-EVs showing a high Pearson’s correlation of multiscatter plots which corroborated the high intragroup similarity within both cohorts: R = 0.87 ± 0.05 for the DLB group and R = 0.82 ± 0.09 for the HC group (Fig. 3A). Aiming to assess possible expression differences between these two cohorts and looking for possible DLB biomarkers, a principal component analysis (PCA) and hierarchical clustering were performed based on the detected proteins. No differential segregation of the two groups was found (Fig. 3B,C).

Focusing now on the analyses of the alternative approach –in gel digestion-, we identified 201 proteins (39.9% of the initially covered) (Fig. 2B) and observed that HCs and DLB shared 167 proteins as analysed by stringent filtering with MaxQuant and Perseus (Fig. 2D). When compared to the first set, only 74 proteins were found in common (Fig. 2B). We found 20 proteins differentially identified in Set 1 compared to Set 2, accounting mainly for immunoglobulins (35%) and individual proteins such as STATH, HLA-A or HLB-B. In the case of Set 2, 126 proteins were specifically found in this analysis compared to Set 1. From those, 13.5% were immunoglobulins, 4% were SERPINS, and 4.8% were complement-related units. Of notice, we identified EV-markers CD81, CD36 and CD9 among these 126 specific proteins of Set 2.

**Figure 4:**
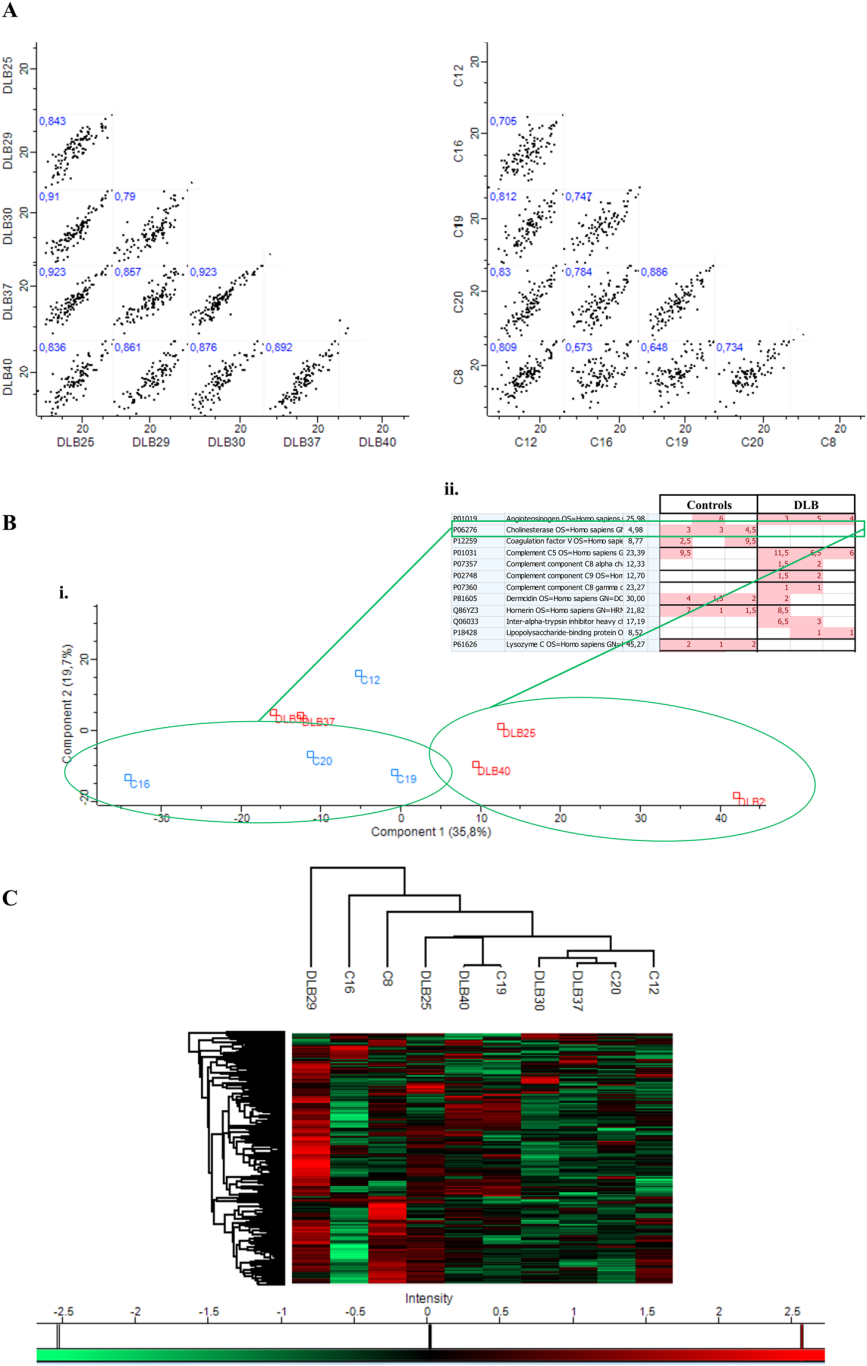
Similarities and differences between cohorts from Set 2. (**A**) Correlation multi-scatter plots gave rise to a Pearson correlation of R = 0.75 ± 0.09 for healthy controls and 0.87 ± 0.04 for DLB samples**. (B**) (**i**) Comparative protein content analysis of both cohorts by PCA showing components 1 and 2, which account for 35.8% and 19.7%, respectively; (**ii**) Butyrylcholinesterase (BCHE) is one of the proteins differentially identified in the two cohorts based on component 1. **(C)** Comparison of protein expression by hierarchical clustering analysis with a heat map of the 201 proteins identified in Set 2.

Again, a high intragroup and intergroup similarity was observed (Pearson correlation: R = 0.87 ± 0.04 for DLB samples with a mean of 132.4 ± 8.4 proteins per sample; and R = 0.75 ± 0.09 for HC samples with an average of 132.8 ± 20 proteins per sample) (Fig. 4A). No defined grouped distribution by PCA and hierarchical clustering analysis was either observed in this second set of samples (Fig. 4B,C) although several samples seemed to segregate (C16, C19, C20 *vs* DLB25, DLB29 and DLB40) based on component 1–35.8%- in PCA (Fig. 4B i). Additional analysis taking into account these six samples revealed proteins differentially found in both groups. Among them, butyrylcholinesterase (BCHE) was identified in 4/5 of HCs and only in one DLB patient. Specifically, it was detected in the 3 control samples from the PCA analysis and in none of the 3 DLB samples that seemed to segregate based on component 1 (Fig. 4B ii). In addition, although in this case, using the second approach, GSN protein was identified in all samples, in line with Set 1, it was highly detected in HCs samples in comparison to the DLB group.

**Figure 5:**
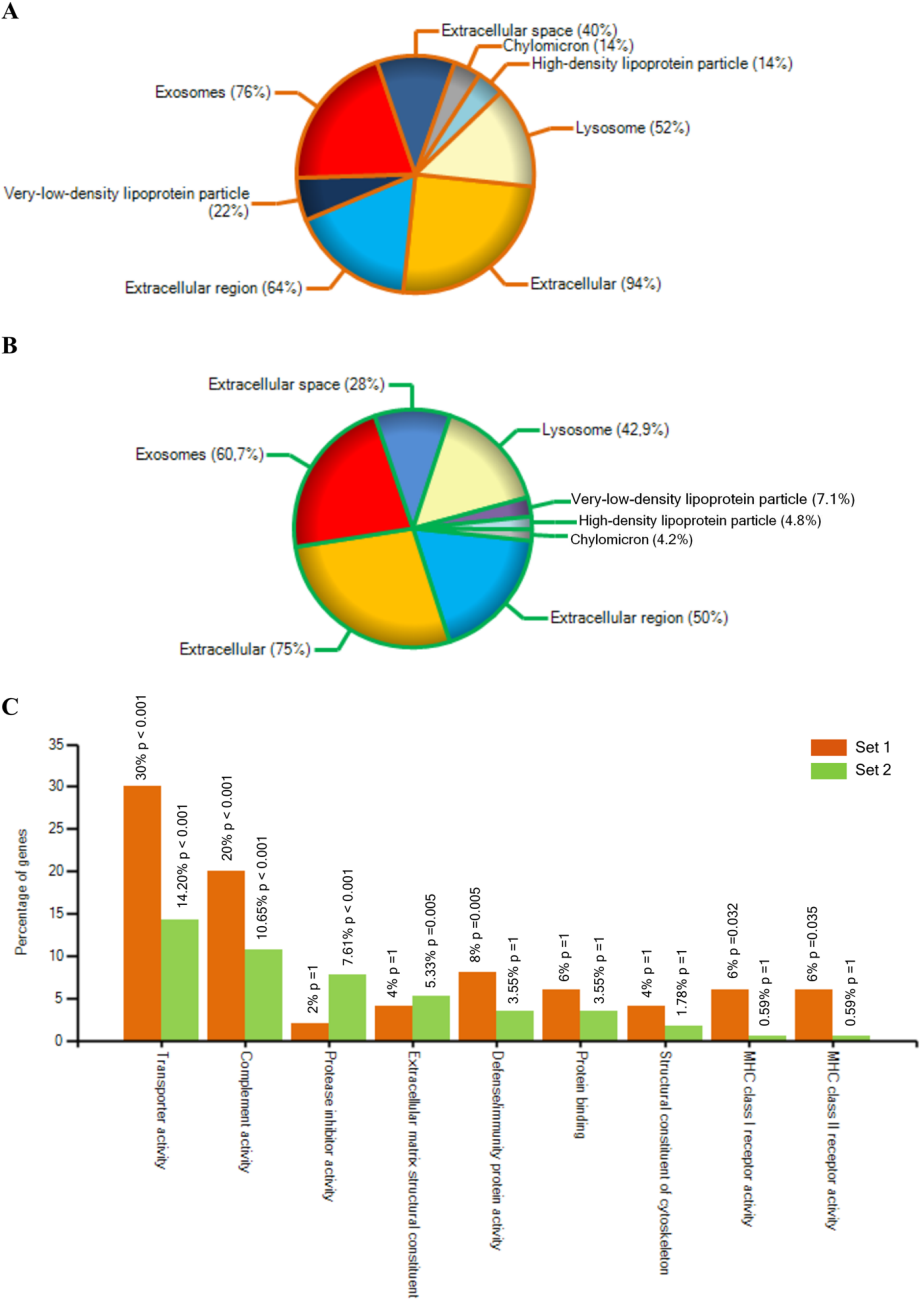
Gene Ontology analysis for the MaxQuant identified proteins in both approaches using FunRich tool^36^. (**A**) Gene Ontology analysis for the cellular component of the proteins found in Set 1. (**B)** Gene Ontology analysis for the cellular component of the proteins found in Set 2. **(C)** Comparative Gene Ontology analysis for molecular function in Set 1 and Set 2. The most over-represented GO terms are shown.

Of notice, GO analysis for cellular component classified the obtained proteome from Set 1 as derived from exosomes (76%), extracellular region (64%) extracellular space (40%) and extracellular (94%) with a p-value < 0.001 (Fig. 5A). Around 50% of all proteins were identified as lipoprotein related. When considering their molecular function, the majority of the identified proteins in this first Set were identified as involved in transporter activity (30%) and immune-related processes such as complement activity (20%) and MHC class I and II receptor activity (12%) (Fig. 5C). Similarly, most of the proteins found using the second approach were identified as exosome component (60.7%), as extracellular region (50%) and space (28%) related by GO for cellular component with a p-value < 0.001 (Fig. 5B). Presence of lipoproteins could also be observed in this set of samples (around 16%). Although in less proportion than in Set 1, proteins identified by the second approach were also related to transporter and complement activity. Of notice, over-representation of proteins related to extracellular matrix constituents and protease activity was found in this second set compared to the first one (Fig. 5C).

Taken together, in both analysed batches, EV-markers were widely identified among the common proteins found in both cohorts, including actin, CD5 antigen-like protein, glyceraldehyde-3-phosphate dehydrogenase, galectin-3-binding protein, moesin and fibronectin (Table 1). Further analyses taking into account the most important EV-protein databases, and their record for “human vesicle protein” were performed. Together, both proteomic approaches identified proteins already described as associated to and/or contained in human EVs (Suppl. Fig. 1).

**Table 1:** Several EV-markers found in our two analyses.

Protein name	Gene symbol	Found in
14-3-3 protein zeta/delta	YWHAZ	Set 1 and Set 2
Actin cytoplasmic 1	ACTB	Set 1
CD5 antigen-like protein	CD5L	Set 1 and Set 2
Glyceraldehyde-3-phosphate dehydrogenase	GAPDH	Set 1 and Set 2
CD81 antigen	CD81	Set 1
Galectin-3-binding protein	LGALS3BP	Set 1 and Set 2
CD9 antigen	CD9	Set 2
Fibronectin	FN1	Set 1 and Set 2
Filamin A	FLNA	Set 1 and Set 2
Apolipoprotein E	APOE	Set 1 and Set 2
Complement C3	C3	Set 1 and Set 2
Clusterin	CLU	Set 1 and Set 2
Apolipoprotein D	APOD	Set 1 and Set 2
Dermcidin	DCD	Set 2
Annexin A2	ANXA2	Set 2
Ficolin 3	FCN3	Set 2
Moesin	MSN	Set 2

Comparison to updated 2018 Vesiclepedia data allowed us to identify different proteins along our shotgun analysis considered among the top 100-EV-markers. Proteins identified in ≥3 of the samples are considered.

**Figure 6:**
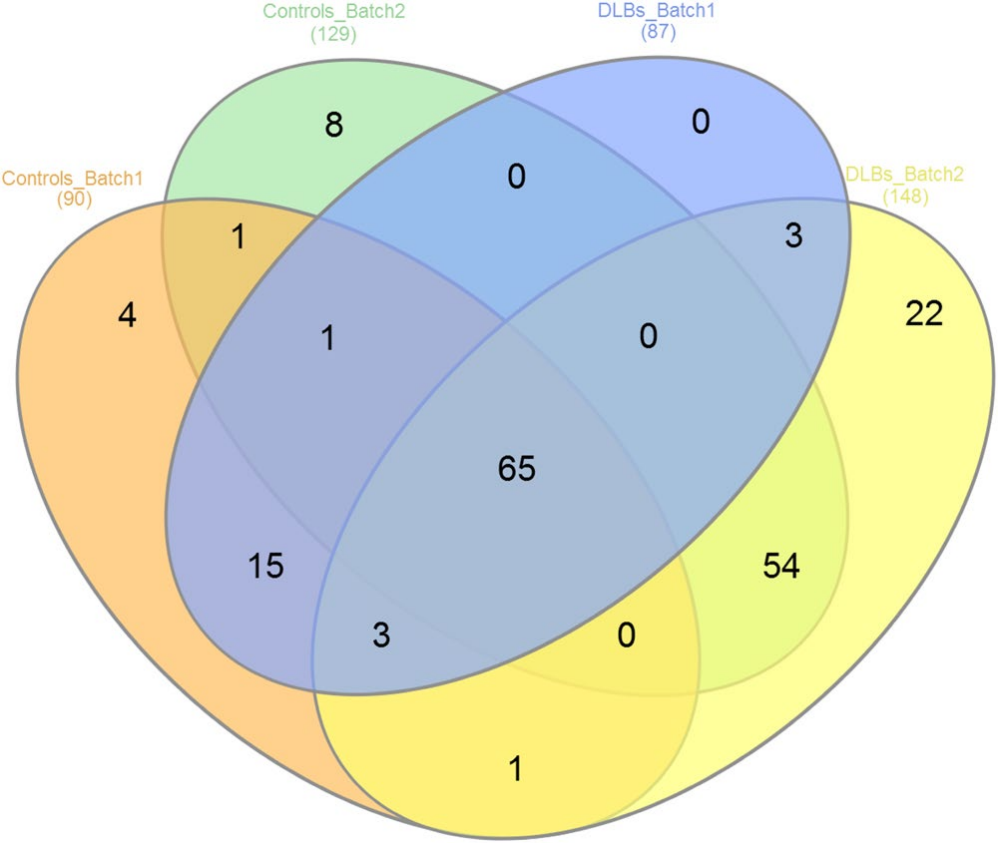
Venn diagram showing the overlap of proteins detected in both MS/MS analysis in the replicated samples. Two DLB-EV samples and two healthy controls EV samples were analysed by both approaches.

Despite the similarities of results in both sets, the duplicated samples submitted to both approaches differed in their protein profile, and samples processed using the in-gel digestion approach rendered a higher number of proteins: 87 proteins were identified in the 2 DLB samples in the in-solution digestion analysis while 148 proteins were found when applying the in-gel digestion. Similarly, the two controls analysed rendered 90 proteins when processed using the in-solution digestion and 128 different proteins when processed in-gel (Fig. 6).

### Validation of possible biomarker capability of selected proteins

Although none of the comparisons revealed major expression differences between DLB and HCs, the particular expression pattern found specifically for GSN and BCHE in the performed analysis was indicative of possible differences. Thus, we further explored the expression of these two proteins using conventional ELISA assays. The concentration of BCHE in all samples analysed was below the detection limit of the assay (data not shown), and thus we did not extend the study of this protein.

**Figure 7:**
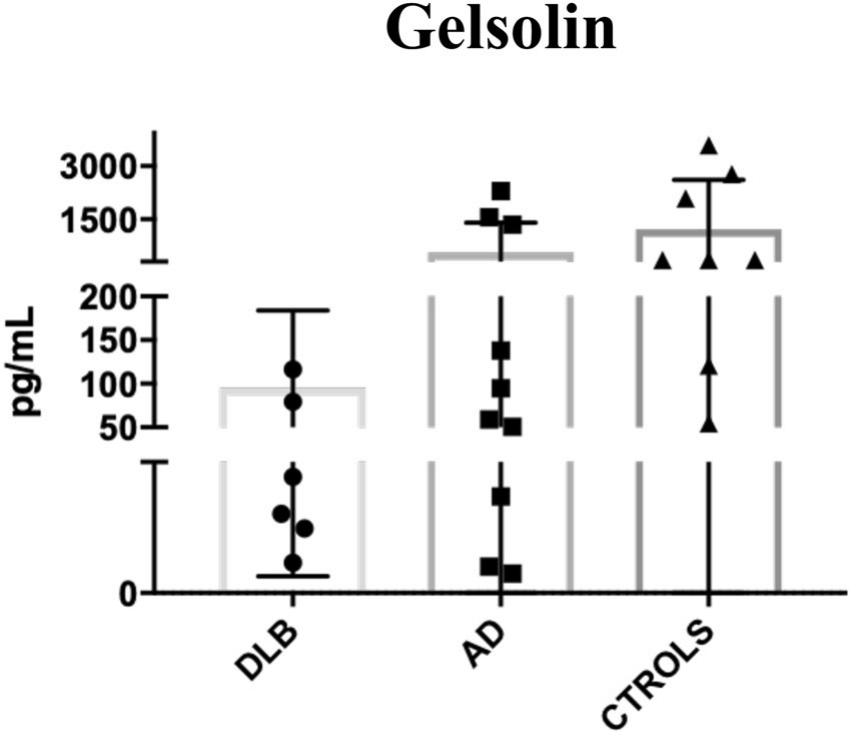
Validation of gelsolin (GSN) as biomarker for DLB. Quantification of GSN levels by ELISA in DLB, AD and controls. Mean ± SD are plotted for each cohort.

With regard to GSN, the ELISA quantitatively confirmed the previous expression pattern observed in the shotgun approach, as it was detected in higher concentration in HCs compared to DLB patients (−21.6 pg/mL to 245.1 pg/mL –mean 95.1 ± 88.7 pg/mL- in the case of DLB patients, compared to −42.5 pg/mL to 3593.9 pg/mL –mean of 1210.8 ± 1397 pg/mL in HC). An additional group of AD patients was included in the assay. Remarkably, GSN was also clearly detected in AD-EV samples (expression levels from 36.8 pg/mL to 1353.4 pg/mL, mean 560.7 ± 845.4 pg/mL) compared to the low expression in DLB (Fig. 7). Nevertheless, probably due to the low number of samples and the high intra-group variability, no statistical significance was reached.

## Discussion

In this study, we aimed to characterize the specific proteomic profile of plasma-derived EVs from patients suffering from DLB as a first step to identify potential minimally invasive biomarkers for this dementia-related disease. During the last years, EVs have emerged as an important source of biomarkers due to the protected environment they provide to their cell-specific molecular content. In this context, for what we think is the first time, we addressed the analysis of the plasma EV-proteome of DLB patients and HCs using two different proteomic approaches. First, an in-solution digestion with LysC and Trypsin enzymes was performed and, in a second batch of samples, an in gel-based analysis followed by Trypsin digestion was applied. Two DLB and two HC samples were duplicated in both sets of analyses as internal controls of the techniques. The comparison of these samples revealed a higher amount of proteins in those processed using the in-gel approach, denoting a possibly higher sensitivity of this technique when working with EV samples. Given the differences found when using these two proteomic approaches, we considered that any consistent result obtained using both technical procedures could be indicative of reliable biomarkers susceptible of further validation.

Aiming to find differences between the healthy and the pathological groups, we analysed separately the two approaches. In both analyses, we found typical markers of EVs, including CD5L, GAPDH or LGALS3BP. Although routinely detected by flow cytometry, no tetraspanin proteins were identified by MS/MS except in some samples from the in-gel approach, as previously reported^37,39^. This observation, together with the detection of other proteins such as Moesin or Ficolin 3 in the second analysis (Table 1), points also to a possibly higher sensitivity for the detection of specific proteins by the in-gel digestion methodology compared to the first in-solution digestion approach. GO analysis showed similar proportion of exosome-, extracellular space- and lysosome- related proteins in both analyses, although higher proportion of lipoprotein co-identification was found among the first set of samples.

When a comparative analysis based on the iBAQ values from MaxQuant was performed, no significant differences were observed between both cohorts. However, two proteins, BCHE and GSN, caught our attention as they showed a specific expression pattern and characteristic distribution between both groups.

The enzyme butyrylcholinesterase (BCHE) is involved in the metabolism of acetylcholine whose deficit is one of the hallmarks of AD and DLB. Recently, a lower BCHE activity was measured in plasma of patients suffering from DLB than in plasma of controls or AD patients^40^, which could be in accordance with the reduced expression of BCHE we have found in plasma-EVs by MS/MS. Nevertheless, using a conventional ELISA we were unable to detect BCHE levels even in the control group.

Gelsolin is an actin-modulating protein that has been described as inhibitor of β-amyloid fibrillation^41^. It has been previously reported as soluble in plasma and cerebrospinal fluid and mutations in its gene cause a systemic amyloidogenic disease and promote AD pathology^42–44^. Moreover, a study on the role of GSN in Lewy body diseases reported the presence of gelsolin together with α-synuclein in Lewy bodies of DLB and PD brains as detected by immunohistochemistry. The same group reported that gelsolin promotes α-synuclein aggregation in the presence of high Ca^2+^ in neuroblastoma cells^45^. Given the appealing expression profile of GSN found in both proteomic approaches, we analysed the presence of this protein in EVs by ELISA in independent cohorts of DLB, HCs and AD patients. ELISA analysis revealed lower levels of GSN in plasma-EVs from DLB and AD patients compared to healthy controls, observing even  much lower levels in DLB samples compared to AD. Hence, although no significant differences were obtained due to the low number of samples and high SD, a clear tendency confirmed the reduced presence of GSN in DLB patients observed by MS/MS. Our results are in accordance with those previously reported for AD patients, which showed lower GSN plasmatic levels compared to control samples^44,46^. A possible speculation could be that lower levels of GSN in plasma-derived EVs could be indicative of dysfunction during AD and DLB, being possibly related to β-amyloid fibril deposition. But, as mentioned, plasma-EVs also reflect a higher impairment of GSN expression in DLB than in AD. The significance of this observation has to be further investigated but it may relate GSN to the specific pathophysiology of DLB and the previously described deposition of GSN in Lewy bodies^45^.

In summary, although a still limited number of patients (which together with the current limitations to discriminate between DLB and AD patients) may explain the lack of statistical significance), the reduced expression of GSN seen in DLB compared to AD suggests that the detection of this protein could be useful as peripheral biomarker.

Additionally, comparison of our data sets and several databases for the proteomic content of vesicles, all of them filtered by human-EV proteins, identified all our obtained proteins as related to EV in any of the databases. Nevertheless, the analysis displayed important differences between the different databases. This may be due, firstly to the lack of consensus of EV-isolation methodology, and secondly, to the different samples (and collection protocols) used. Moreover, not all the data sets can be obtained applying the same filters to different the databases. Efforts should be made to unify and standardise, not only the EV-related methodology but also the data recovering of EV-related studies.

## Conclusions

In conclusion, we provide a description of the plasma-EV proteome from aged patients suffering from DLB and its comparative analysis to healthy controls using two different proteomic approaches. Our results pointed to in-gel digestion-based methodology as a more sensitive method to identify proteins in samples with low protein content, such as SEC-derived EVs. Interestingly, one of the two proteins identified as putative biomarkers by these “shotgun” proteomic approaches (GSN) was validated by ELISA and the results showed a clear tendency of decreased gelsolin concentration in DLB-EVs compared to controls-EVs and, importantly, to AD patients. This observation must be further confirmed in a larger cohort of patients and with different protein-detection assays.

## Supplementary information

Supplementary Figure 1: Qualitative comparison of the identified proteins with EV-data bases.
