# Supplementary material for: Comprehensive proteomic profiling of plasma-derived Extracellular Vesicles from dementia with Lewy Bodies patients
Source: Sci Rep. 2019 Sep 16;9:13282. doi: 10.1038/s41598-019-49668-y (PMC6746766; doi:10.1038/s41598-019-49668-y)
Supplement: Supplementary file 1 — Supplementary Figure 1: Qualitative comparison of the identified proteins with EV-data bases. [file 41598_2019_49668_MOESM1_ESM.pdf]

Comprehensive proteomic profiling of plasma-derived Extracellular Vesicles from dementia with Lewy Bodies patients

Ana Gámez-Valero[1, 2], Jaume Campdelacreu[5], Ramón Reñé[5], Katrin Beyer[1†*] and Francesc E. Borràs[2, 3, 4†*]

[1]Department of Pathology, Hospital Universitari and Health Sciences Research Institute Germans Trias i Pujol, Universitat Autònoma de Barcelona, Spain.

[2]REMAR-IVECAT group, Health Sciences Research Institute Germans Trias i Pujol, Badalona, Spain.

[3]Nephrology Service, Hospital Universitari Germans Trias i Pujol, Badalona, Spain.

[4]Department of Cell Biology, Physiology and Immunology, Universitat Autònoma de Barcelona (UAB), Barcelona, Spain.

[5]Department of Neurology, Hospital Universitari de Bellvitge, L'Hospitalet de Llobregat, Spain.

[†]**Both senior authors contributed equally.**

***Address correspondence** to

Katrin Beyer, PhD, Department of Pathology, Hospital Universitari Germans Trias i Pujol, 08916 Badalona, Barcelona, Spain. Phone: 00-34-93-497 88 53, e-mail: katrinbeyer@hotmail.com

Francesc E. Borràs, PhD, REMAR-IVECAT group, Health Sciences Research Institute Germans Trias i Pujol, 08916 Badalona, Spain. Phone: 00-34-93-497 86 71, e-mail: feborras@igtp.cat

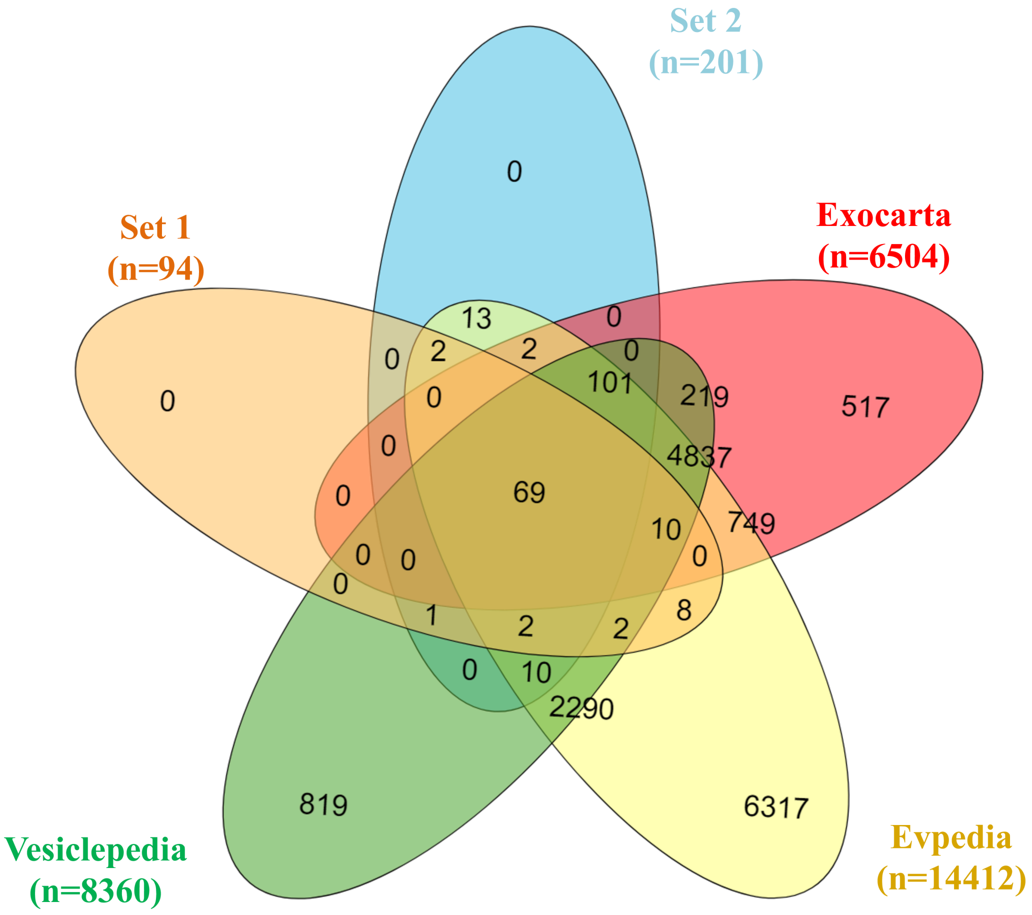

Supplementary Figure 1: Qualitative comparison of the identified proteins with EV-data bases. Venn diagram showing the overlap of proteins detected among the proteins found in both sets of samples and those reported in ExoCarta, EVpedia and Vesiclepedia as EV-human related (2018).
